# 50nm-Scale Localization of Single Unmodified, Isotopically Enriched, Proteins in Cells

**DOI:** 10.1371/journal.pone.0056559

**Published:** 2013-02-19

**Authors:** Anthony Delaune, Armelle Cabin-Flaman, Guillaume Legent, David Gibouin, Caroline Smet-Nocca, Fabrice Lefebvre, Arndt Benecke, Marc Vasse, Camille Ripoll

**Affiliations:** 1 Laboratoire MERCI EA3829, équipe AMMIS, Faculté des Sciences de l’Université de Rouen, Mont Saint Aignan, France; 2 Faculté de Médecine-Pharmacie de l’Université de Rouen, Rouen, France; 3 Institut des Hautes Etudes Scientifiques & CNRS USR3078, Bures sur Yvette, France; Charité-Universitätsmedizin Berlin, Germany

## Abstract

Imaging single proteins within cells is challenging if the possibility of artefacts due to tagging or to recognition by antibodies is to be avoided. It is generally believed that the biological properties of proteins remain unaltered when ^14^N isotopes are replaced with ^15^N. ^15^N-enriched proteins can be localised by dynamic Secondary Ion Mass Spectrometry (D-SIMS). We describe here a novel imaging analysis algorithm to detect a few ^15^N-enriched proteins - and even a single protein - within a cell using D-SIMS. The algorithm distinguishes statistically between a low local increase in ^15^N isotopic fraction due to an enriched protein and a stochastic increase due to the background. To determine the number of enriched proteins responsible for the increase in the isotopic fraction, we use sequential D-SIMS images in which we compare the measured isotopic fractions to those expected if 1, 2 or more enriched proteins are present. The number of enriched proteins is the one that gives the best fit between the measured and the expected values. We used our method to localise ^15^N-enriched thymine DNA glycosylase (TDG) and retinoid X receptor α (RXRα) proteins delivered to COS-7 cells. We show that both a single TDG and a single RXRα can be detected. After 4 h incubation, both proteins were found mainly in the nucleus; RXRα as a monomer or dimer and TDG only as a monomer. After 7 h, RXRα was found in the nucleus as a monomer, dimer or tetramer, whilst TDG was no longer in the nucleus and instead formed clusters in the cytoplasm. After 24 h, RXRα formed clusters in the cytoplasm, and TDG was no longer detectable. In conclusion, single unmodified proteins in cells can be counted and localised with 50 nm resolution by combining D-SIMS with our method of analysis.

## Introduction

Molecules present in relatively low numbers in the cell often play a disproportionately important role for the phenotype, a phenomenon known as minority control [1] . Analytical biochemistry generally entails extraction from many cells and hence yields information on the average concentration/amount. To obtain information on the role of rare macromolecules in biological variability due, for example to stochastic effects, requires single cell analysis [2].

Cellular imaging allows rare macromolecules to be localised in single cells but this generally entails labelling [3]. By “labelling” we mean here the process of linking to the target macromolecule a group of atoms that can be detected using a physical technique. Labelling of target macromolecules may perturb their biological activity; for example, a large fluorescent tag, like GFP, may have steric or other effects that alter the properties of the labelled macromolecule [4]–[5]. Antibody recognition of a macromolecule may be prevented if the epitope is masked by folding or by interactions between the macromolecule and other molecule, or indeed, if the antibody itself is attached to a fluorescent tag [6]. Detection by fluorescence of rare macromolecules may be prevented if the specific signal is masked by, for example, the autofluorescence background.

Label-free imaging of macromolecules within the single cell continues to make major advances. Raman scattering and coherent anti-Stokes Raman scattering provide images of chemical bonds even in label-free molecules [7]. However, without labelling, these techniques do not allow one protein to be distinguished from another within a cell since all proteins have the same chemical bonds. Cryo-electron tomography allows the label-free imaging of a single protein in cells [8] but, to achieve this result, it requires a perfect knowledge of the tertiary structure of the protein to exploit its electron scattering pattern. Hence, it is possible neither to distinguish between two proteins that have only slight differences in structure nor to identify proteins of unknown folding. Mass spectrometry-based imaging techniques allow the sample material itself to be analysed directly rather than its interaction with photons or electrons; these techniques include Matrix-Assisted Laser Desorption and Ionization imaging which is currently limited to a 10 µm resolution and is therefore unsuitable for intra-cellular localization on the sub-micrometer scale [9]. Mass spectroscopic methods also include imaging using either static or dynamic Secondary Ion Mass Spectrometry (SIMS), which can attain sub-micrometer resolution and which are therefore suitable for the intra-cellular localization of chemically unmodified molecules within single cells [10].

Dynamic Secondary Ion Mass Spectrometry (D-SIMS) is one of the most sensitive analytical imaging techniques [11]–[13]. In D-SIMS, a high fluency primary ion beam (*e.g.* with 16 keV Cs^+^ ions) is focussed as a *microprobe* on a region of the surface of a sample for a chosen *dwell time* to generate fragments of molecules from the sputtered volume corresponding to a pixel; we term this volume a *sputtel*. Some of these fragments, which are either atoms or small molecular clusters, are sputtered out from the surface as charged *secondary* ions that are then separated in a mass spectrometer. This allows the simultaneous counting of several different species of ions (up to five or seven in the current state of the art) from the sputtel.

The beam is rastered across the surface to generate an image of X×X pixels (e.g. X = 128 or 256) for each secondary ion selected. In these images, the grey level (or false colour) for each pixel (i, j) represents the number of counts of the imaged secondary ion in the corresponding sputtel (i, j) where i and j are the coordinates of the position of the pixel in the image (i and j are in the range [0, X−1]). A new sample layer can then be sputtered out to give a new image with exactly the same set of i and j coordinates as the previous image but at a different depth. Repeating the process yields a series of images originating from a series of successively sputtered layers (ν = 1, 2, … ν_max_) of the sample. Therefore the position of a pixel or the corresponding sputtel in a given layer is written (i, j)[ν]. The lateral resolution of the images provided by SIMS depends on the size of the microprobe and on the number of counts. Modern imaging instruments like the CAMECA NanoSIMS 50 allow a lateral resolution down to a few tens of nm [14]–[15].

D-SIMS has the apparent drawback for imaging cells of being an elemental analysis. In an elemental analysis, the carbon atoms, for example, counted in a sputtel cannot be attributed readily to a single species of macromolecule because they are also constituents of many other molecules and macromolecules present in the same sputtel. However, SIMS can discriminate not only between elements but also between isotopes such as ^12^C and ^13^C. Hence SIMS can discriminate between a specific macromolecule enriched in one or more rare stable isotopes such as ^13^C, ^15^N, ^2^H, ^34^S, ^18^O, etc. (or even isotopes that have a long half-life such as ^14^C) and the normal, non-enriched, macromolecules. Note that D-SIMS imaging is a label-free technique in the sense that it is only based on a difference in isotopic composition and is not based on chemical modifications such as iodination or tagging with fluorophores like GFP. In a sputtel that contains only a few, highly enriched, *small molecules* and many non-enriched molecules, the number of rare isotopes extracted from the enriched molecules may be much less than the number of rare isotopes extracted from the non-enriched molecules; in this case, statistically significant detection may be impossible. In a sputtel that contains a few enriched *macromolecules*, however, D-SIMS fragments them into tens, hundreds, or even thousands of isotope-enriched ions, which gives a number of counts much greater than those coming from the non-enriched molecules and thus greatly facilitating detection.

Here we report the combination of D-SIMS and a new algorithm to reliably detect within a cell a *single*, isotopically enriched, macromolecule. As test macromolecules, we have chosen thymine DNA glycosylase (TDG), which exists only as monomers, and retinoid X receptor α (RXRα), which exists mainly as dimers and tetramers. These macromolecules were ^15^N-enriched, purified and delivered into COS-7 cells. Using a NanoSIMS 50, we obtained quantitative, isotopic images of these cells, in which we determined the number of ^15^N-enriched proteins and their distribution on the 50 nm scale.

## Results

### Expected Increase in the ^15^N Isotopic Fraction Due to the Presence of ^15^N-enriched Proteins in the Sputtel (the Volume Sputtered Corresponding to a Pixel

In D-SIMS of biological samples, sputtered nitrogen and carbon atoms recombine in the form of CN^–^ secondary ions [11] [16]–[19]. These are detected with a high useful yield (ratio of the number of detected atoms to the number of sputtered atoms of a given species). There are 2 stable isotopes of carbon and nitrogen; therefore there are 4 isotopic species of CN secondary ions: ^12^C^14^N, ^13^C^14^N, ^12^C^15^N and ^13^C^15^N. In samples containing the carbon and nitrogen isotopes in their natural abundances, the relative percentages of these secondary ions are: 98.530, 1.104, 0.362, and 0.004 respectively [20].

In any region of our samples, the number of carbon atoms is much higher than the number of nitrogen atoms. Therefore, for each pixel (i, j) the ^12^C^15^N isotopic fraction r_ij_ = n_ij_(^12^C^15^N)/[n_ij_(^12^C^15^N)+n_ij_(^12^C^14^N)] is a measure of the local ^15^N isotope fraction. In this expression, n_ij_(^12^C^15^N) and n_ij_(^12^C^14^N) are the numbers of counted ^12^C^15^N and ^12^C^14^N secondary ions, respectively, obtained from the sputtel. We calculated (see Text S1, section ***“Case of a single spherical ^15^N-enriched protein”***) that the presence of a single spherical ^15^N-enriched protein in the sputtel increases the local ^15^N isotopic fraction by a factor up to 5, depending on the relative amounts of the enriched protein and the non-enriched molecules that are sputtered (Figure S1).

**Figure 1:**
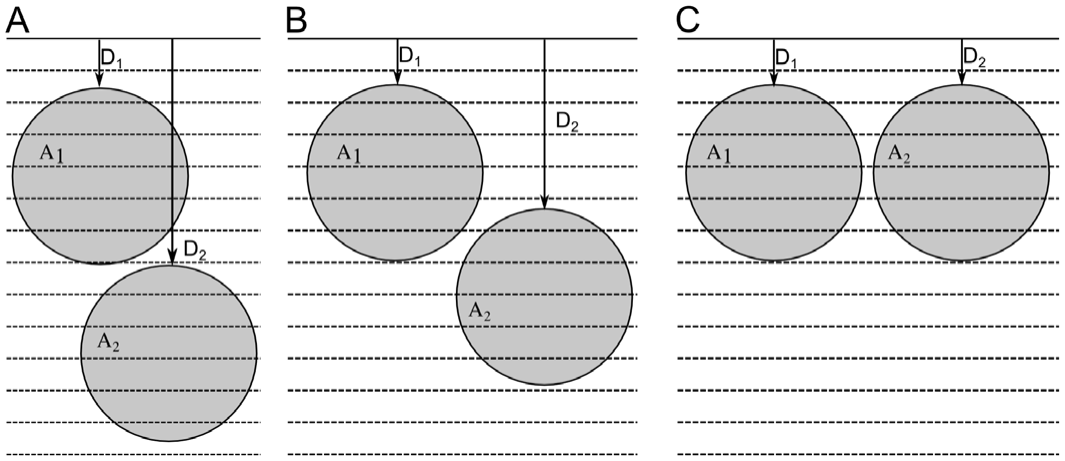
Positions of 2 identical proteins (5.6 nm in diameter) A_1_ and A_2_ relative to the sample surface. The top of the first protein A_1_ is located at a distance D_A1_ = 1.5 nm from the initial sample surface. 13 sputtered layers, 1 nm in thickness, are represented. (A): the top of the second protein A_2_ is at D_A2_ = 7.1 nm from the surface (i.e. D_A2_ = D_A1_+5.6 nm). (B): the top of the second protein A_2_ is at D_A2_ = 5.45 nm (i.e. D_A2_ = D_A1_+5.6×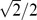 nm). (C): the top of the second protein A_2_ is at D_A2_ = D_A1_ = 1.5 nm.

**Figure 2:**
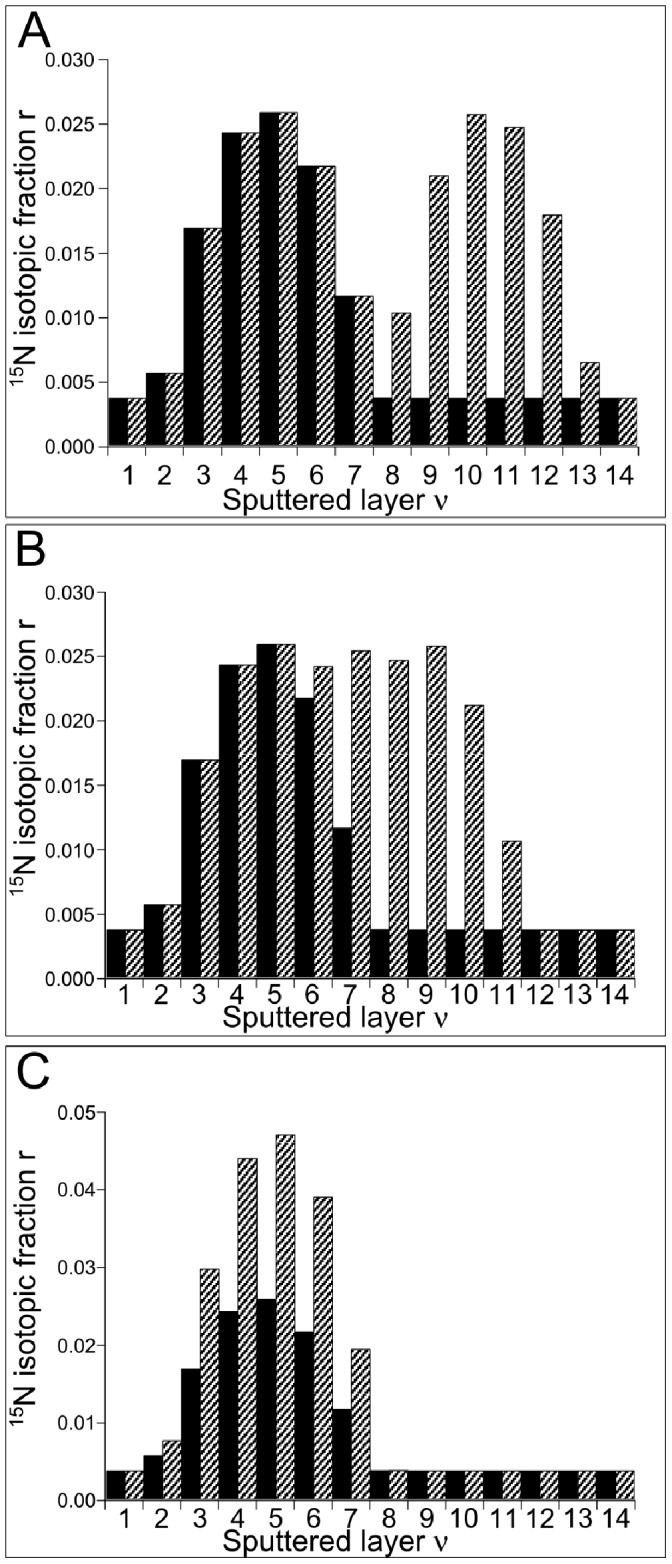
Calculated values of the ^15^N isotopic fraction in the successive sputtered layers of the 2-protein models represented in **Figure 1A, 1B and 1C** respectively (shaded bars). For comparison the values of the ^15^N isotopic fraction obtained with a single enriched protein located at a distance D = D_A1_ from the initial sample surface are also indicated (bars in filled black). Note that in Figure 2B, each of the sputtels in layers 4 to 9 has the same value of the ^15^N isotopic fraction although the sputtels in layers 4 and 5 only contain a segment of protein A_1_ (and similarly for A_2_ in 8 and 9) whereas the sputtels in layers 6 and 7 contain segments of both proteins (see **Figure 1B**).

In principle, these relative amounts could be used to decide how many enriched proteins are present in a region provided account is taken of the position of these proteins with respect to the successive layers of sputtering (which give rise to pixels with the same i, j coordinates in each layer ν) as shown in Figure 1. This figure shows sputtels containing two identical spherical proteins of diameter d, A_1_ and A_2_. Protein A_1_ is at a distance D_A1_ from the surface and protein A_2_ is at a distance D_A2_≥D_A1_. In the particular case where the two proteins are in contact to one another (dimer), the axis of the dimer (straight line joining the two protein centres) forms an angle α with the initial (before sputtering) sample surface and D_A2_ = D_A1_+d×sin(α). Since the two proteins are in the same sputtel, the ^15^N isotopic fraction of the sputtel is independent of whether the proteins are in contact or not. Three positions of the two proteins are shown in Figure 1: D_A2_ = D_A1_+d (Figure 1A), D_A2_ = D_A1_+d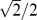 (Figure 1B) and D_A2_ = D_A1_ (Figure 1C), corresponding, in the particular case of a dimer, to angles α = 90, 45 and 0°, respectively. We have calculated the ^15^N isotopic fraction in the successive sputtels (as explained for a single protein in Text S1) and the results are shown in Figure 2A, 2B and 2C corresponding to the proteins at the positions shown in Figure 1A, 1B and 1C, respectively. In the case where D_A2_ = D_A1_+d (Figure 2A), the ^15^N isotopic fractions of the sputtels first increase in the layers 1 to 5 and then drop in the layers 5 to 7; exactly the same values are obtained with a single protein; in layer 8, however, in the single protein case, the ^15^N isotopic fraction of the sputtel reaches the natural value 0.0037 whereas in the case of two proteins this value remains 0.0102 and then increases over the next few sputtels. It is therefore possible to distinguish between the presence of one and the presence of two enriched proteins in the series of sputtels. In the case where D_A2_ = D_A1_ (Figure 2C), the isotopic fractions in the sputtels of successive layers 1 to 5 increase and then drop progressively to the natural value in layers 6 to 8. This increase is approximately twice that obtained for a single enriched protein. It is therefore again possible to determine if one or two enriched proteins were present in the series of sputtels. A particularly interesting scenario is shown in Figure 1B. Suppose that the first image acquired corresponds to the sputtering of the 5^th^ layer (Figure 2B), if the sample contains a single enriched protein, the ^15^N isotopic fractions measured in successive sputtels are 0.0257, 0.0215, 0.0115 and 0.0037 whereas if the sample contains two enriched proteins with D_A2_ = D_A1_+d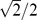, the isotopic fractions are 0.0257, 0.0240, 0.0252 and 0.0244. In other words, for one protein the values drop progressively over the four sputtels to the natural value whereas for two proteins the values do not drop significantly over the four sputtels. Figure 2 therefore shows that the sputtering of only one layer is insufficient to distinguish between one and two proteins in the sputtel. However, acquisition of a sequence of four images obtained from the sputtering of four successive sample layers should, in principle, allow one and two proteins to be distinguished.

In the above calculations we have assumed that the enriched proteins are spherical. Similar calculations can be performed for proteins with different shapes to show that if the enriched proteins are completely sputtered, their shape does not affect their estimated number (Text S1, section *“Case of one or several non-spherical ^15^N-enriched proteins”* and Figures S2 and S3).

### Algorithm to Localise ^15^N-enriched Proteins

#### Statement of the problem

In the above calculations of the ^12^C^15^N isotopic fraction r_ij_ = n_ij_(^12^C^15^N)/[n_ij_(^12^C^15^N)+n_ij_(^12^C^14^N)] statistical fluctuations in the n_ij_ counting were ignored. Such fluctuations in ^12^C^14^N and ^12^C^15^N counts can be described by a Poisson distribution law. Evidence for this distribution has been obtained in the analysis of different samples using either static SIMS [21]–[23] or dynamic SIMS [24]–[25]. We verified, using a Kolmogorov-Smirnov test, that the ^12^C^14^N and ^12^C^15^N counts in homogeneous areas of our non-enriched samples (*e.g.* embedding resin) indeed obeyed a Poisson distribution (not shown). Therefore even in a perfectly homogeneous sample (of uniform ^15^N isotopic fraction) the measured r_ij_ values are distributed with a given variance relative to the mean. Hence a stochastically *high* value in the counting n_ij_(^12^C^15^N) of ^12^C^15^N and/or a stochastically *low* value in the counting n_ij_(^12^C^14^N) of ^12^C^14^N may result in a substantial r_ij_ increase that may be confused with the presence of an enriched protein (false positive). Reciprocally, statistical fluctuations that result in a substantial r_ij_ decrease that may lead to the wrong conclusion that an enriched molecule is absent when it is actually present (false negative). This clearly shows the need for a statistical criterion that allows one or several enriched proteins in the sputtel to be detected with a chosen probability (e.g. the risk of a false positive, α, equals 0.05). We therefore developed an algorithm to calculate the probability distribution laws for the n_ij_(^12^C^14^N), n_ij_(^12^C^15^N) and for the resulting isotopic fraction r_ij_ as explained in Text S2. In the following, a sputtel that contains one or more ^15^N-enriched proteins is termed an “enriched sputtel” and the corresponding pixel is termed an “enriched pixel”. Conversely, a sputtel that does not contain ^15^N-enriched proteins is termed “non-enriched sputtel” and the corresponding pixel is termed a “non-enriched pixel”. The set of non-enriched molecules contained in the sputtel determines the background value for the ^15^N (natural) isotopic fraction.

#### Statistical criterion for determination of the enrichment status of a pixel

Using our algorithm to obtain the probability distribution law for the background ^15^N isotopic fraction (Text S2), we obtain a map of (p-values)_ij_ of the sputtels selected from the image. This p-value is the probability that the measured ^15^N isotopic fraction r_ij_ is an observation of the *background* ^15^N isotopic fraction. Because sputtels with the lowest p-value might be enriched, a threshold p-value (p-value)_thres_ is chosen such that if (p-value)_ij_ ≤ (p-value)_thres_ then the pixel (i, j) is considered as enriched with a risk α of being a false positive. The classical Bonferroni correction for multiple testing permits a (p-value)_thres_ to be determined with an associated α risk:
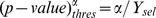
where Y_sel_ is the number of pixels selected from an image (Text S2). The Bonferroni correction, however, is known to be stringent and thus increases the risk of false negatives. Therefore we decided to use two thresholds with associated risks α = 0.05 and α = 0.1, i.e. 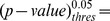
0.05/Y_sel_ and 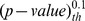 = 0.1/Y_sel_. For example in an image of 128×128 pixels the number of selected pixels is typically 10000. Hence 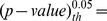 5×10^–6^ and 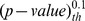 = 10^−5^. Hence we define two classes of enriched pixels. The first class includes pixels (i, j) that are considered as enriched since:



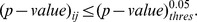



The second class includes pixels that are considered as enriched since:
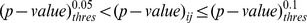



Using these two thresholds to analyse more than 150 images of control cells (cells that have not been in contact with ^15^N enriched molecules) we found no enriched pixels, although an analysis of so many images at an α risk of 0.05 should have given a few images with false positives. This suggests that our method reduces the risk of false positives. That said, our image analysis carries an increased risk, albeit low, of false negatives. We have preferred to run the risk of the false negatives in order to ensure our method for localising proteins really does work.

The ^12^C^14^N images are displayed in grey levels (these images reveal intracellular structures). The pixels excluded by the initial threshold and pixels discarded after running the algorithm are displayed in black (Text S2). The enriched pixels of the first class are displayed in blue and those of the second class are displayed in red. Data files are also created including the estimated Poisson parameter λ (Text S2) and p-value for each pixel analysed.

### Algorithm for Determination of the Number of Enriched Proteins in a Sputtel

One problem in deciding whether one or two proteins are present in a sputtel is that the same value for the ^15^N isotopic fraction can be obtained in both cases as explained above (first section of “Results”). To help solve this problem, we have shown that the number of proteins in a sputtel can be determined by acquiring a sequence of SIMS images from successive layers whatever the shape of these proteins. This problem is, however, compounded in measurements of real samples because of the statistical fluctuations in the isotopic fraction. We describe here an algorithm to compare the values of the ^15^N isotopic fraction measured in a sequence of sputtered layers to the values obtained from different models. Each of these models is defined by the number of proteins of a particular shape and size. Varying the model parameters (e.g. the distance of the proteins from the surface) permits the algorithm to determine the model that best fits the experimental observations.

The algorithm, inspired from the classical optimization methods in numerical analysis, comprises the following steps:

1. We consider an enriched pixel (i, j) present in a series of ν_max_ images (successive layers). For this pixel the experimental data are the thicknesses, e_ij_[ν] (estimated as explained in Text S3 and illustrated in Figure S4 via the ^16^O counts in the pixel) and the values of the ^15^N isotopic fractions r_ij_[ν] of the successive sputtels (i, j)[ν] (with ν = 1,…, ν_max_).
2. We choose an initial model that is thought to reasonably fit the data: e.g. a single spherical protein of known diameter or 2 cylindrical proteins.
3. We calculate for this model the values of the ^15^N isotopic fractions, r^model^[ν], that would be obtained after sputtering the successive layers of the estimated thicknesses (Text S1).
4. We then calculate a “distance” κ between the ^15^N isotopic fractions calculated for the model and those obtained experimentally
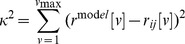

5. We vary stepwise the parameters of the model until the lowest value of κ, termed κ_min_, is reached and the corresponding determination coefficient R^2^ is calculated.
6. We then choose another plausible model (e.g. with a different number or shape of proteins) and repeat steps 3 to 5.
7. Finally, we choose the best model by comparing the κ_min_ and R^2^ values and by directly examining the individual residuals (the differences between the measured ^15^N isotopic fractions and the theoretical values given by each model).

### Application to COS-7 Cells Transfected with TDG and RXRα Proteins

#### Example of high-resolution localisation of TDG and RXRα ^15^N-enriched proteins

**Figure 3:**
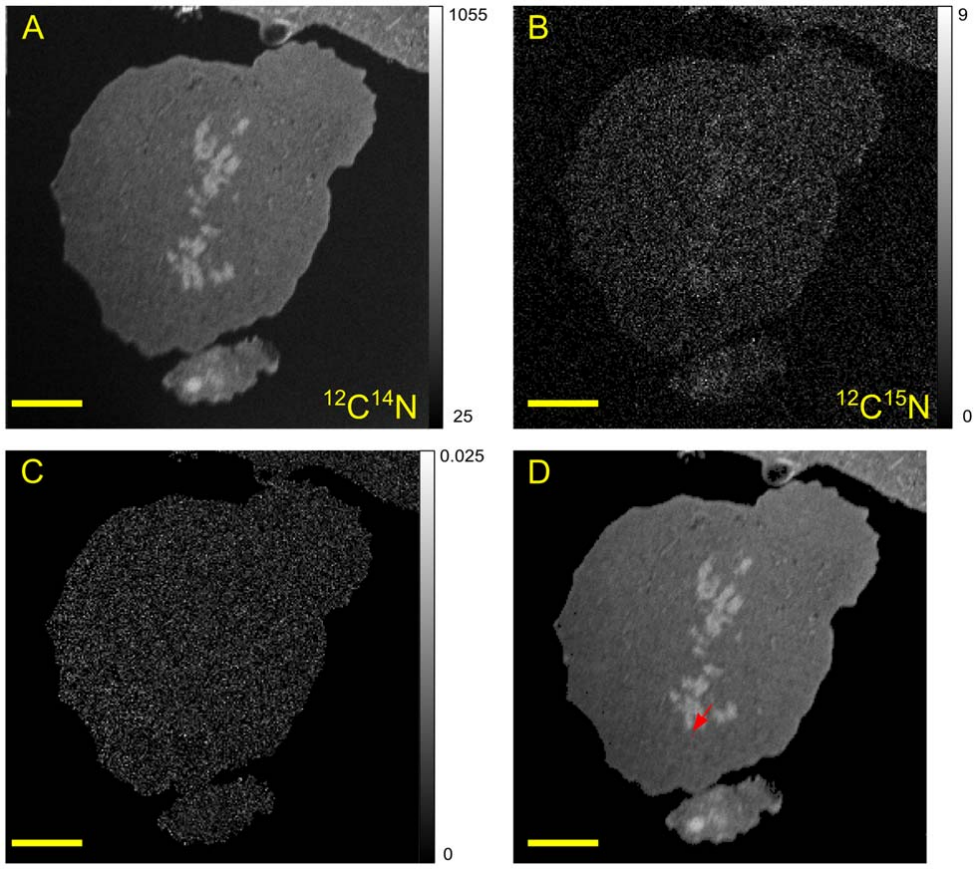
SIMS images of a COS-7 cell after 4 h incubation with ^15^N-enriched TDG/Provectin. The primary current was 1 pA and the dwell time 15 ms. (A): 256×256 pixels image of ^12^C^14^N, (B): 256×256 pixels image of^ 12^C^15^N, (C): 250×250 pixels image of the isotopic fraction ^12^C^15^N/(^12^C^15^N+^12^C^14^N), (D): 250×250 pixels image of ^12^C^14^N with one enriched pixel displayed in red (arrow). Scale bar is 5 µm. The corresponding grey level scale is shown on the right of the image.

COS-7 cells are derived from the kidneys of Green monkeys, which resemble human kidneys, and are considered to be easy to transfect. ^15^N-enriched human TDG proteins were introduced into COS-7 cells using the Provectin delivery reagent. High-resolution images were obtained of a cell, which is probably in late prophase, showing the distribution of ^12^C^14^N and ^12^C^15^N secondary ions, and of the ^15^N isotopic fraction (Figure 3). The ^12^C^15^N image (Figure 3B) is similar to the ^12^C^14^N (Figure 3A) but, as expected, with larger statistical fluctuations due to a lower number of counts. Using our algorithm we found one enriched pixel of the second class (p-value = 3.10^−6^ and r_ij_ = 0.0250) as shown in red in Figure 3D.

**Figure 4:**
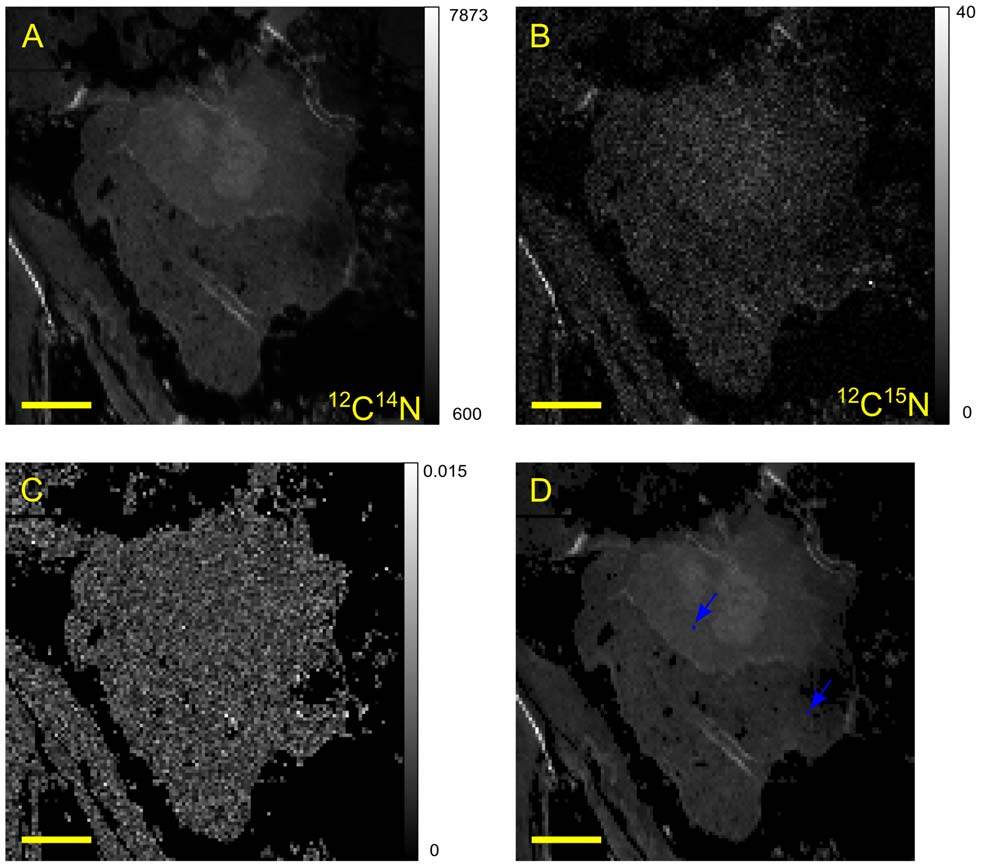
SIMS images of a COS-7 cell after 4 h incubation with ^15^N-enriched RXRα/Provectin. The primary current was 2 pA and the dwell time 150 ms. (A): 128×128 pixels image of ^12^C^14^N, (B): 128×128 pixels image of^ 12^C^15^N, (C): 122×122 pixels image of the isotopic fraction ^12^C^15^N/(^12^C^15^N+^12^C^14^N), (D): 122×122 pixels image of ^12^C^14^N with enriched pixels displayed in blue (arrows). Scale bar is 5 µm. The corresponding grey level scale is shown on the right of the image.

^15^N-enriched mouse RXRα proteins were also introduced into COS-7 cells using the Provectin delivery system (Figure 4). Using our algorithm to analyse the ^15^N isotopic fraction (Figure 4C), we found two enriched pixels of the first class (as shown in blue in Figure 4D) which, coincidentally, have the same p-values (4.10^−6^). The first enriched pixel (r_ij_ = 0.0119) is located in the nucleus whilst the second is in the cytoplasm (r_ij_ = 0.0144).

A total of 178 images of TDG- or RXRα-transfected cells (using Provectin) were analysed; 28 showed enriched pixels of the first class and 44 showed enriched pixels of the second class. The remaining 106 images showed no enriched pixels. To investigate whether this absence of enriched pixels was due to a poor delivery into COS-7 cells by the Provectin system, we used this method to introduce fluorescent “control IgG” into these cells. Flow cytometry showed that 60% of the cells did not contain the IgG and that the remaining 40% contained around 3000 IgG. Assuming that the 3000 IgG in a COS-7 cell are homogeneously distributed, an entire sputtered slice of the cell, 5 nm thick, should contain on average one IgG. With a Poisson law of parameter 1, this result suggests that there should be 0, 1, 2, 3 and 4 enriched pixels in 37, 37, 18, 6 and 2% of the images, respectively. This result is consistent with our SIMS observations.

#### Example of quantification of TDG and RXRα ^15^N-enriched proteins

**Figure 5:**
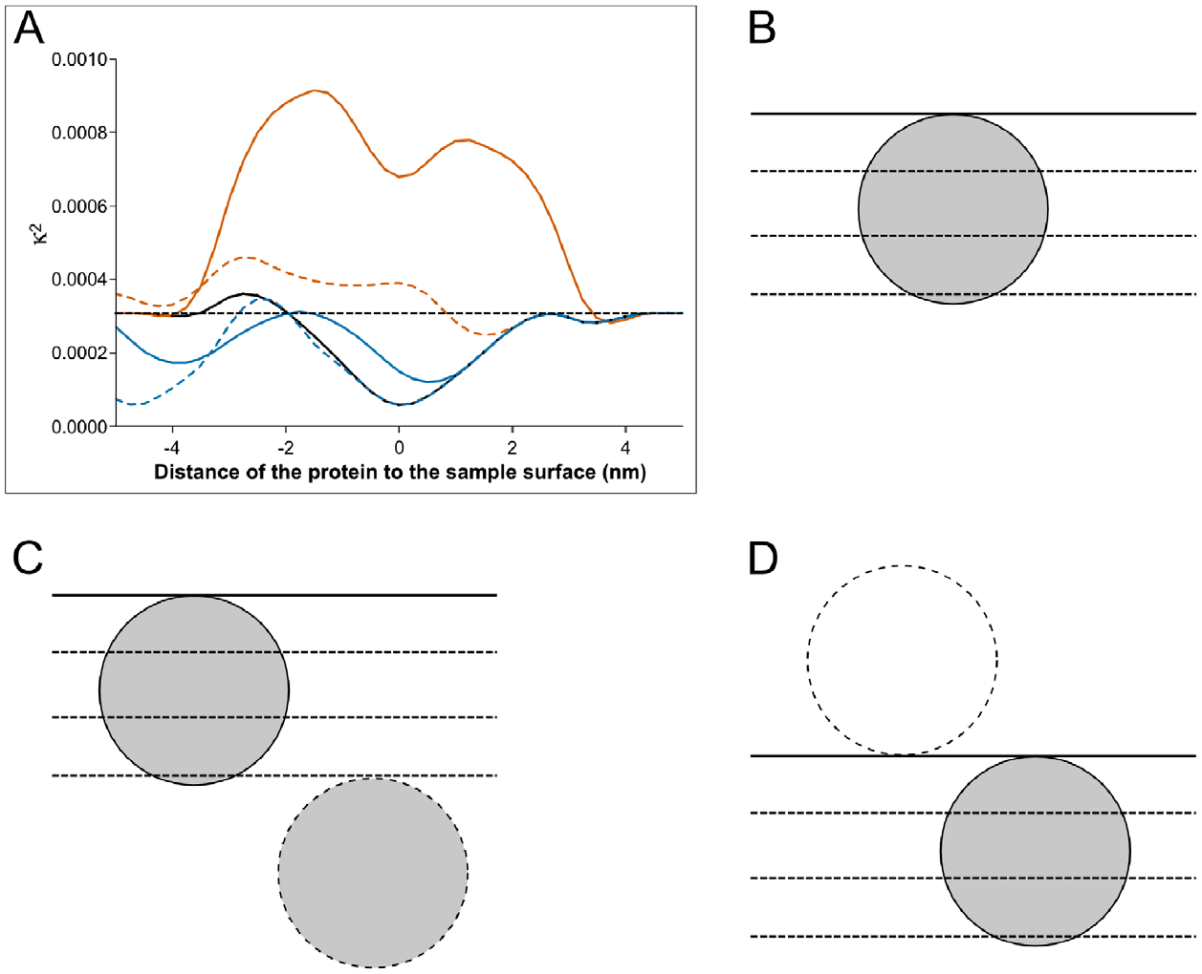
Quantification of ^15^N-enriched TDG proteins (diameter d = 4.7 nm). A sequence of 3 successive SIMS images of a COS-7 cell transfected with TDG/Provectin was acquired for the same field of view (27×27 µm). The primary current was 1 pA and the dwell time 20 ms. (A): variation of κ^2^ as a function of the distance D for the 1-protein model (curve in black) and 2-protein model with D_A1_ = D and D_A2_ = D_A1_+d (dashed curve in blue), D_A2_ = D_A1_+d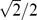 (curve in blue), D_A2_ = D_A1_+d/2 (dashed curve in vermilion) and D_A2_ = D_A1_ (curve in vermilion). The black dashed horizontal line represents the value of κ^2^ in absence of enriched protein. (B): representation of the protein position in the case of the best fit 1-protein model. (C): representation of the protein position in the case of the best fit 2-protein model. (D): best fit 2-protein model; trivial case where one of the 2 proteins was eliminated during the processes preceding the image acquirement. This arrangement corresponds to the minimum of κ^2^ at D = –4.7 nm for the dashed curve in blue of panel (A).

As explained above, the measurement of the ^15^N isotopic fraction in a series of sequential images can be used to determine the number of enriched proteins. Consider first the TDG protein. The values of the measured ^15^N isotopic fraction r_82,189_[ν] and the corresponding sputtered thickness e_82,189_[ν] (using the number of counts of ^16^O) in 3 successive layers (images) are given in Table 1 for three pixels with the same coordinates (i = 82, j = 189). Using these isotopic fractions, our algorithm identified the pixel in the second layer of sputtering as being enriched but not the pixels in the first and third layers. This enriched pixel (82,189) [2] was of the second class. Assume that there is a single enriched protein in the corresponding sputtel (82,189) [2]. Figure 5A shows the variation of κ^2^ = 
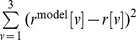
 as a function of D. Clearly the best fit is obtained for a distance D = 0 nm with the corresponding κ^2^_min_ value of 0.0000575; the calculated r^model^[ν] values are given in Table 1 and the best fit model is shown in Figure 5B. The curves in Figure 5A show that a 2-protein model gives a κ^2^_min_ value equal to that of a 1-protein model only when D_A1_ = D = 0 and D_A2_≥ e_82,189_ [1]+e_82,189_ [2]+e_82,189_ [3] = 4.49 nm (Table 1) or D_A2_≤ − d = − 4.7 nm; hence the sputtered layers contained only one of the two proteins (Figure 5C and 5D). When – d ≤ D_A2_≤4.49 (Figure 5A) the κ^2^_min_ values are much greater than that corresponding to that of the 1-protein model. These results suggest that a single enriched protein is present in the sputtels. That said, the determination coefficient R^2^ = 0.2174 of the “1-protein” model makes this interpretation questionable and it might be argued that. the measured r_82,189_[ν] values might simply be due to statistical fluctuations occurring in the absence of enriched proteins (the “0-protein” model). We reject the “0-protein” model because: 1) the “distance” κ^2^ = 0.000308 between the “0-protein” model and the experiment is an order of magnitude greater than “distance” between the “1-protein” model and the experiment (see dotted line in Figure 5A), 2) there is a low probability of statistical fluctuations that give substantially increased values of the background isotopic fraction in each of 3 successive sputtered layers. It should be noted that one reason that the determination coefficient of the “1-protein” model may be rather low would be if the TDG protein had been degraded (see the following section) and only a part of the native protein was present in the sputtels (mainly in layer 2).

**Table 1:** Example of quantification of TDG protein in pixel (82,189)[ν].

Layer ν	Measuredr_82,189_[ν]	Thicknesse_82,189_[ν](nm)	Best fit model: 1 proteinr^model^[ν]
1	0.0066	1.46	0.0122
2	0.0202	1.59	0.0201
3	0.0088	1.44	0.0140

In conclusion, even in this difficult case, our method permitted the detection of a single enriched protein.

**Figure 6:**
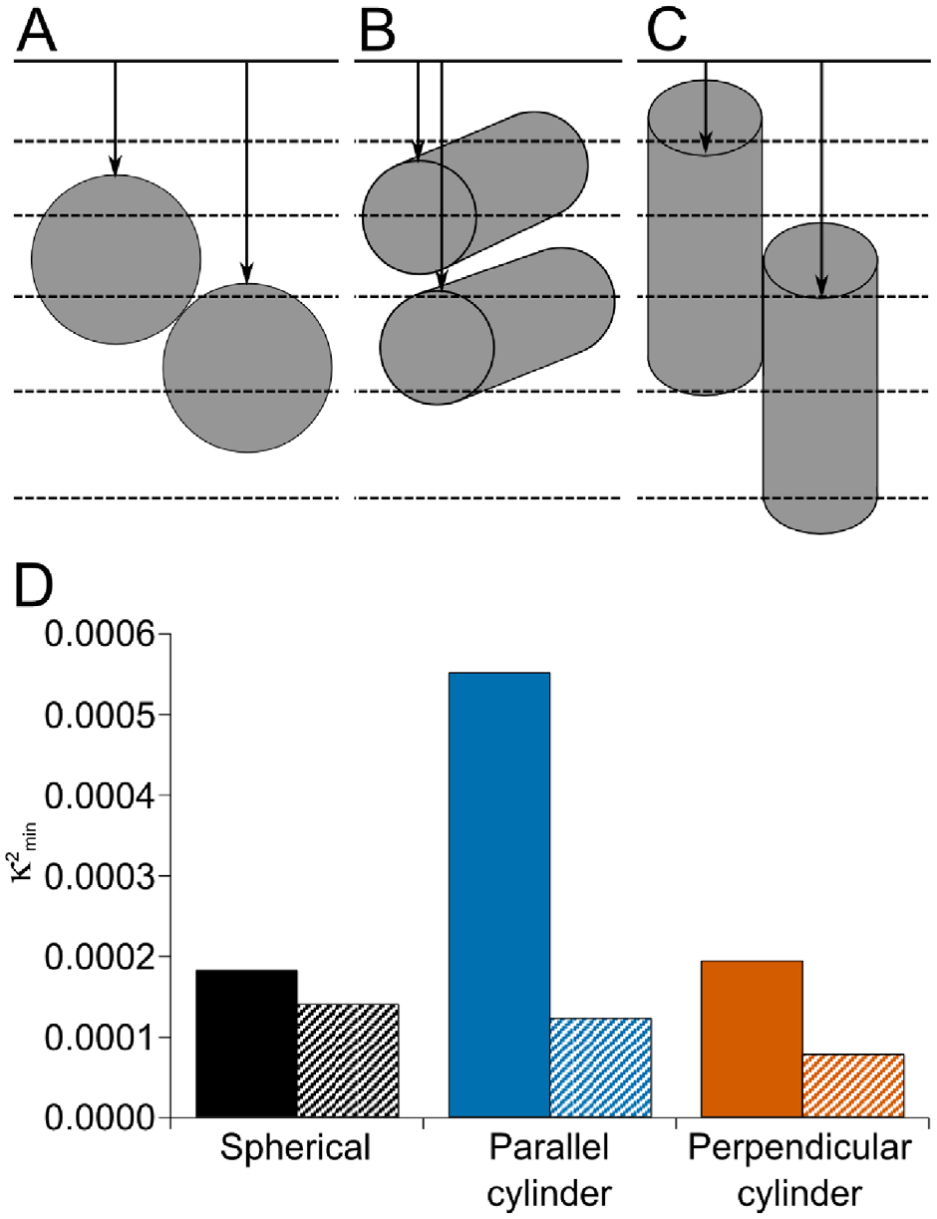
Quantification of ^15^N-enriched RXRα proteins. A sequence of 5 successive SIMS images of a COS-7 cell transfected with RXRα/Provectin was acquired for the same field of view (17×17 µm). The primary current was 1.2 pA and the dwell time 60 ms. (A): the model with two spherical proteins that best fits the experimental isotopic fractions. (B) and (C): the models with two cylindrical proteins that best fit to experimental isotopic fractions, long axis of the cylinders parallel (B) or perpendicular (C) to the initial sample surface. (D): Values of κ^2^_min_ for the 1-protein (filled bars) and 2-protein (shaded bars) models represented in (A), (B) and (C).

Consider now the RXRα protein. In this example we measured the ^15^N isotopic fraction r_61,113_[ν] together with the sputtered thickness e_61,113_[ν] in 5 successive layers (Table 2). Here, our algorithm detects the pixels (i = 61, j = 113) of layers 2 to 4 as enriched pixels of the first class whereas pixels of layers 1 and 5 are considered as non-enriched. The RXRα protein was purified and delivered into cells as a fusion GST-RXRα protein; unfortunately the tertiary structure of this fusion protein is unknown. We therefore modelled it both as a sphere and as a cylinder, with its long axis either parallel or perpendicular to the initial sample surface. For each of these shapes we varied the number of enriched proteins and their distance from the sample surface. Figures 6A, 6B and 6C show the positions of the proteins corresponding to the best fit models for 2 spherical proteins, 2 cylindrical proteins with their long axes parallel to the sample surface and 2 cylindrical proteins with their long axes perpendicular to the sample surface, respectively. Figure 6D gives the κ^2^_min_ values of the 1-protein (filled bars) and 2-protein (shaded bars) models shown in Figure 6A, 6B and 6C. Whatever the shape and orientation of the enriched proteins, the 2-protein models always gave lower κ^2^_min_ values than the one-protein models. The calculated r^model^[ν] values for the 1-protein and the 2-protein best fit models are given in Table 2. In the 2-protein model that gave the best fit, the enriched proteins are cylinders perpendicular to the sample surface. The r^model^[ν] values are close to the measured r_63,113_[ν]. In contrast, we found that for a 1-protein model, the best fit is obtained for a spherical protein. With this model, however, there is no enriched protein in layer 2 whilst we measured a statistically significant enrichment r_63,113_ [2], hence we rejected this model.

**Table 2:** Example of quantification of RXRα protein.

Layer ν	Measured r_63,113_[ν]	Thickness e_63,113_[ν] (nm)	Best fit model: 1 protein r^model^[ν]	Best fit model: 2 proteins r^model^[ν]
1	0.00365	2.61	0.00366	0.00366
2	0.01061	2.42	0.00366	0.01353
3	0.01404	2.65	0.01859	0.01483
4	0.02801	3.09	0.01786	0.02433
5	0.00672	3.48	0.00366	0.01413

Taken together, these results show that there were 2 ^15^N-enriched proteins in the sputtels (63,113).

#### Fate of TDG and RXRα enriched proteins as a function of time

**Figure 7:**
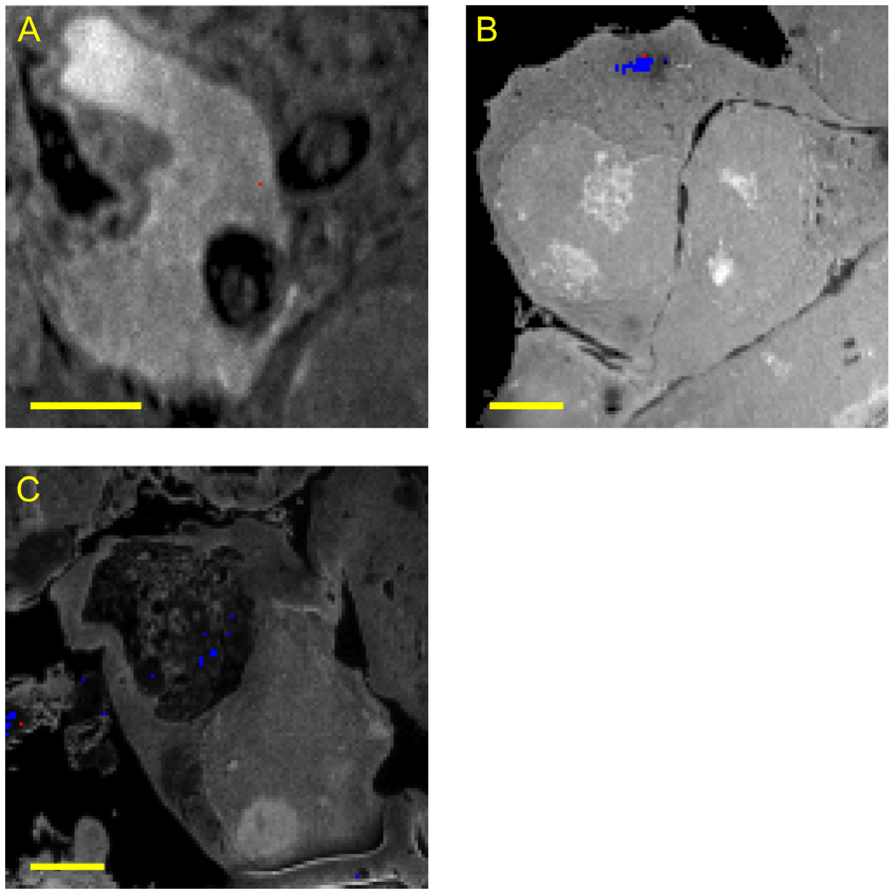
^12^C^14^N SIMS images of COS-7 cells transfected with RXRα (A) and (C) or TDG (B). (A) and (B): 7 h incubation, (C): 24 h incubation. Bar = 5 µm. Primary current = 1 pA (A) or 1.5 pA (B) and (C); dwell time = 15 ms (A), 100 ms (B) or 150 ms (C). Detected enriched pixels are superimposed either in blue (first class) or in red (second class enriched pixel).

Representative locations of TDG (Figure 3) and RXRα (Figure 4) are shown after 4 hours incubation with the provectin system. At this time, the pixels adjacent to an enriched pixel were never themselves enriched i.e. the enriched pixels were isolated. Usually, these enriched pixels were in the cell nucleus. Moreover, our quantification method showed that the enrichment most likely corresponded to the presence of a single or two enriched proteins. We never found an isotopic enrichment that corresponded to more than two enriched proteins. After 7 hours incubation, TDG was mainly outside the nucleus in large clusters spread over a few contiguous pixels (Figure 7B); at 7 hours incubation, isolated monomers, dimers and tetramers of RXRα were visible in the nucleus but no clusters of RXRα were visible in the cytoplasm (Figure 7A). After 24 hours incubation, small clusters of RXRα were found together in a structure reminiscent of an autophagic vacuole (Figure 7C). After 24 hours incubation, TDG could no longer be detected, consistent with the protein having been degraded. Together these observations suggest that our method may be used to give information on the kinetics of turnover of proteins.

## Discussion

The enrichment of macromolecules with one or more rare stable isotopes of its constituent elements, such as ^13^C and ^15^N, provides a powerful way to facilitate their detection in D-SIMS. Detection is favoured by reduction of the size of the sputtel containing the isotopically enriched macromolecules, since this decreases the number of non-enriched molecules in the sputtel (background) and therefore increases the signal to background ratio. This can be achieved by reducing the diameter of the primary beam, which also improves the spatial resolution, down to the limit imposed by the need to have sufficient secondary ions to count.

Flooding of neutral caesium on the sample surface increases significantly the useful yield of secondary ions [26]–[28]. The use of caesium flooding therefore increases the capacity of SIMS to detect single enriched molecules even if these molecules are small. Previously, we have used the combination of caesium flooding and SIMS to detect DNA [29] but, as shown here, other species of biological macromolecules can be detected providing they contain isotopes in sufficient numbers that are different from the background.

One advantage of isotopic enrichment is that “native” macromolecules can be studied without the need for tags [4] [30]. Such enrichment entails using rare, stable isotopes of the elements that constitute the macromolecule. These isotopes are present in a low proportion in the unenriched macromolecules and therefore generate a background SIMS signal that can interfere with the detection of the enriched molecules. The secondary ion used in this work is CN^-^ which is mainly a recombinant molecular ion [11] [16]–[18]; the recombination process is local and occurs under the beam between atoms a few nm apart in the sample [19]. Hence, a single protein enriched with both ^15^N and ^13^C might be detected using the recombinant ^13^C^15^N^-^ since the background level of this ion is very low. A radioactive isotope with a long half-life such as ^14^C could also be used [31]. In this case, the background level of ^14^C is almost zero and this increases substantially the signal to background ratio; moreover, caesium flooding might be used to improve the detection of carbon directly by increasing its ionisation yield.

In a previous study [19], we have shown that the formation of the recombinant ^13^C^15^N secondary ion between a ^13^C- enriched macromolecule and a ^15^N-enriched neighbouring macromolecule could be used as a basis for their co-localization. This technique could be extended to the colocalisation of a macromolecule enriched with ^15^N and another with ^14^C using the recombinant ^14^C^15^N secondary ion and the algorithm described here.

The response of neuroblastoma cells to retinoic acid is under active study in our laboratory and elsewhere [32]. The exact role(s) of the RXRα receptors involved in this response, linked perhaps to different locations, is unknown. There are relatively few of these receptors in cells and we therefore took on the challenge of using the method of isotopic enrichment and NanoSIMS 50 analysis to try to quantify and to localise them with a resolution of a few tens of nm. RXRα is known to exist as active homo or heterodimers [33] or inactive tetramers [34]. After 4 or 7 h incubation with isotope-enriched RXRα, we only found monomers or dimers of RXRα within cells, consistent with the isotope-enriched protein being in an active state within the nucleus. After 24 h incubation, clusters of RXRα were observed in the cytoplasm that may correspond to degradation structures resulting from the introduction of too many copies of RXRα and/or the introduction method itself which uses cationic lipids that may perturb the cell. After a short incubation time of 4 h, we found TDG in the nucleus where it was always in a monomeric form, as expected [35]. After 7 h, TDG was no longer detected in the nucleus but formed clusters in the cytoplasm; after 24 h, TDG was no longer detected anywhere in the cell, presumably due to its degradation.

Our SIMS-based technique of single protein localisation has the important advantage of using unmodified proteins. It does, however, have the apparent disadvantage of requiring the introduction of isotopically enriched proteins into cells. The technique may therefore prove particularly valuable to the study of systems in which molecules are delivered naturally; such systems include, for example, the study of infection of host cells by bacteria, viruses and prions; internalisation after receptor-ligand interactions, and bacterial conjugation. In some cases, however, artefacts generated by the protein delivery process may be a source of concern. One way to avoid delivering proteins exogeneously would be to enrich the protein of interest *in vivo*. This might be achieved by site-directed incorporation of isotope-enriched, non-natural amino acids in the protein using modified tRNA synthetases [36]–[39] as in NMR studies [40]. To obtain statistically significant detection, use may be made of multi-isotope enrichment (e.g., using both ^14^C and ^15^N as discussed above) of non-natural amino acids and/or the binding to these non-natural amino acids of atoms with a high useful yield in SIMS (such as Br or I). Detection would then require only a slight modification of the protein if caesium flooding is also used to increase the useful yield. The method might then play for D-SIMS a role similar to that played for optical microscopy by the use of fluorescence tags. In addition, the method would offer minimal perturbation of cells, high resolution and a low limit of detection.

## Methods

### Production of ^15^N-enriched RXRα and TDG

The RXRα protein was expressed in *Escherichia coli* BL21 (DE3) strain transfected with the pGEX-full length GST-RXRα plasmid (under the control of the T7 promoter, Lac operator). Briefly, bacteria were grown for 4 days in liquid M9 minimal medium [2 mM MgSO_4_, 0.1 mM CaCl_2_, 0.2% (w/v) glucose, 6 g/L Na_2_HPO_4_, 3 g/L KH_2_PO_4_, 0.425 g/L NaCl and 1 g/L U-^15^NH_4_Cl (Isotec/Sigma-Aldrich) supplemented with 100 mg/L ampicilin] at 37°C and 230 rpm orbital shaking. Bacteria were then inoculated into fresh medium and protein synthesis was induced by 0.5 mM isopropyl-beta-thiogalactoside when the OD600 reached 0.7. Three hours after induction at room temperature, bacteria were harvested, pelleted, resuspended in a lysis buffer [20 mM Tris pH 7.9, 150 mM NaCl, 20% glycerol, 0.1% NP40, 5 mM β-mercaptoethanol, 0.5 mM PMSF, 1× protease inhibitor cocktail (Sigma) and 100 µg/mL lysozyme] and sonicated twice on ice. The supernatant was cleared by centrifugation and incubated with gluthathione-agarose beads (Sigma), previously equilibrated in the GST-buffer [50 mM Tris pH 7.9, 150 mM NaCl, 5% glycerol, 0.1% NP40, 1 mM EDTA, 1 mM dithiothreitol, 0.5 mM PMSF and 1× protease inhibitor cocktail], for 4 hours at 4°C. Beads were then washed three times with GST-buffer and the RXRα protein was eluted with the same buffer but containing 1 mM glutathione.

We measured the ^15^N isotopic enrichment of the bacterial proteins (including RXRα) as follows. We deposited small droplets (approximately 0.5 µL) on the surface of a single face-polished silicon wafer (Siltronix Archamps, France) cleaned using Piranha mixture (50% hydrogen peroxide/sulphuric acid). The ^15^N isotopic ratio was measured using the NanoSIMS 50; we found for RXRα: 0.94±0.02 (mean±standard deviation from 6 replicates).

^15^N-enriched TDG protein was produced and purified as previously described [41]. The ^15^N enrichment (>98%) of the TDG protein was measured by MALDI-TOF mass spectrometry.

### Cell Culture

COS-7, green monkey kidney cells, were grown on Dulbecco’s modified Eagle’s medium (Eurobio, Les Ulis, France) supplemented with 5% fetal calf serum, 2 mM L-glutamine, 100 µg/mL streptomycin and 100 U/mL penicillin. The total nitrogen content of COS-7 was measured by the Kjeldahl method. Briefly, 2.10^7^ cells were washed thrice with PBS [137 mM NaCl, 2.7 mM KCl, 4.3 mM Na_2_HPO_4_, 1.4 mM KH_2_PO_4_, pH 7.2], added to 20 mL of concentrated H_2_SO_4_ and heated until total mineralization (approximately 2 hours). The solution was then alkalinized with an excess of NaOH and ammoniac was distilled in a 10 mL 0.05 M H_2_SO_4_ solution and titrated with a 0.1 M NaOH solution.

### Delivery of the TDG or RXRα Proteins into the Cells

Cells were seeded on a Melinex film (Agar Scientific, Saclay, France) in a culture dish one day prior the introduction of TDG or RXRα proteins. TDG and RXRα proteins were delivered into the COS-7 cells with Provectin-Imgenex protein delivery reagent (Cliniscience, Montrouge, France) according to the manufacturer’s protocol. Briefly, dry Provectin was hydrated for 5 minutes with a 0.2 g/L solution of TDG or RXRα proteins in PBS and then diluted in culture medium without serum. Cells were washed with PBS and the medium containing protein/Provectin was added to the cells and incubated for 4, 7 or 24 hours. Serum was added to a final concentration of 5% only in the case of incubations longer than 4 hours.

### Flow Cytometry

Control IgG1-FITC (9.6 FITC per antibody according to the supplier Santa Cruz Biotechnology, Heidelberg, Germany) were delivered in COS-7 cells using the same method as for TDG and RXRα. After 4 or 7 hours of incubation with IgG1-FITC/Provectin cells were washed thrice with PBS and harvested using cell dissociation solution (Sigma). Cell suspensions were analysed using an EPICS XL flow cytometer (Beckman-Coulter, Villepinte, France). The percentage of positive (fluorescent) cells and the average intensity of FITC signal were measured using 10^4^ control (without IgG1-FITC) or incubated cells. The average intensity of FITC signal was calibrated using fluorospheres (Dako, Trappes, France) giving an average number of FITC per cell for each condition. This number is simply divided by 9.6 to obtain the number of IgG1-FITC per cell.

### Sample Preparation for Sims Imaging

Cells were washed thrice with PBS, fixed for 1 hour at 4°C with a 2% paraformaldehyde, 2% glutaraldehyde, 3.5% sucrose in PBS, washed again thrice with PBS. Cells were then dehydrated in successive baths of ethanol-water: for 5 min each in 30%, 50%, 60%, 70% (v/v), for 10 min in 80%, 90% and 10 min twice in 100%. Pure ethanol was then replaced for 4 hours by a mixture 1∶2, then 2∶1, of ethanol and soft London Resin White (Agar Scientific) and finally replaced by pure resin monomers for 4 hours (renewed twice). Resin was polymerized overnight at 60°C. Semi-thin sections 300 nm in thickness, were made using a diamond knife (Diatome, Biel, Switzerland) mounted on an Ultracut microtome (Leica Microsystems, Rueil-Malmaison, France) and deposited on a P-doped silicon wafer (Siltronix, Archamps, France). Two different batches of resin were used for the preparation of cells transfected with RXRα or TDG (and their corresponding controls, i.e. without introduction of enriched proteins). The preparations of TDG samples were carried out 12 months after those of RXRα.

### Sims Imaging

Samples were analyzed with the multicollection image mode of a NanoSIMS 50 ion analyzer (Cameca, Gennevilliers, France). Briefly, the NanoSIMS 50 was used in the negative secondary-ion mode with the Cs^+^ primary ion beam. High spatial resolution 256×256 pixels images were obtained using a primary beam 1 pA in intensity, D1–3 diaphragm (approximate beam diameter 60 nm) and a dwell time of 20 ms/pixel. For better counting statistics per pixel, 128×128 pixels images were acquired with a primary beam 2 pA in intensity, using the D1–2 diaphragm (approximate beam diameter 150 nm) and a dwell time of 150 ms/pixel. Secondary ions were energy-filtered in order to obtain a mass resolution of 5000 minimum (10% height peak measurement). The images of the ^12^C, ^16^O, ^12^C^14^N, ^12^C^15^N and ^31^P distributions were simultaneously acquired.

### Image Processing and Statistical Analysis

The detection algorithm, written in C language, was run on the raw NanoSIMS images. All calculations were performed on the numbers of counts associated to each pixel of the image. Results were saved as text files. Graphic representation was obtained by importing text data files using ImageJ software and exported as an uncompressed image file. When a pixel was considered as enriched, calculations for quantification were carried out using the Maple 9.5 software (Maplesoft, Waterloo, Canada).

## Supporting Information

Figure S1 Calculated values of the ^15^N isotopic fraction in a series of sputtered layers including part of a single enriched protein. (A): Representation of a sequence of sputtered layers. The initial surface of the sample (before sputtering) is chosen as the origin of the depth axis z, positive downward. The successive sputtered layers, each of thickness e, are numbered 1, 2, …ν, … from the initial sample surface. The top of a spherical enriched protein (diameter d) is located at an algebraic distance D from the origin of the depth axis. r_top_ is the radius of the upper base of the spherical segment of protein in the sputtered layer, r_bottom_ the radius of the lower base and h, the height of the segment (here h = e in each layer except in layers 1, 2 and 7). (B): ^15^N isotopic fraction in each successive sputtered layer represented in (A). Indeed in layer 1, which does not contain enriched protein, the isotopic fraction is equal to its natural value 0.00366.
(TIF)

Figure S2 Representation of two cylindrical proteins. (A): the long axis of the proteins is parallel to the initial sample surface. (B): the long axis of the proteins is perpendicular to the initial sample surface. The size of the cylindrical protein is characterized by the length of its long axis L_c_ and the radius of its section a. The distances of the two proteins relatively to the initial sample surface, D_1_ and D_2_, are defined as the algebraic minimal distances between each protein and the initial sample surface.
(TIF)

Figure S3 Calculated values of the ^15^N isotopic fraction in a series of sputtered layers including enriched proteins. These values were calculated for the presence of one (filled bars) or two (shaded bars) ^15^N-enriched proteins assumed as cylinders with their long axis either parallel (blue bars) or perpendicular (vermilion bars) to the sample surface. The length of the long axis of the cylinder, L_c,_ is equal to 8 nm and the radius, a, is equal to 1.9 nm. The top of proteins is located at a distance D_1_ = D_2_ = 1.5 nm from the sample surface. For comparison, values calculated for the presence of a single spherical ^15^N-enriched protein (5.6 nm in diameter and located at D = 0.7 nm from the sample surface) are shown (black bars). Each sputtered layer is 1 nm in thickness and (π/4) ×(100)^2^ nm^2^ in surface.
(TIF)

Figure S4 Mean value of the number of ^16^O secondary ions per pixel as a function of the estimated mean sputtered thickness of the sample. The straight line is the regression line.
(TIF)

Text S1 Detailed calculations of the expected increase in ^15^N-isotopic fraction due to the presence of ^15^N-enriched proteins in the sputtered volume corresponding to a pixel (sputtel).
(DOC)

Text S2 Algorithm for the determination of the probability distribution law of the background ^15^N-isotopic fraction.
(DOC)

Text S3 Method for the determination of the sputtered thickness.
(DOC)
