# Supplementary figures and images for: 50nm-Scale Localization of Single Unmodified, Isotopically Enriched, Proteins in Cells
Source: PLoS One. 2013 Feb 19;8(2):e56559. doi: 10.1371/journal.pone.0056559 (PMC3576336; doi:10.1371/journal.pone.0056559)

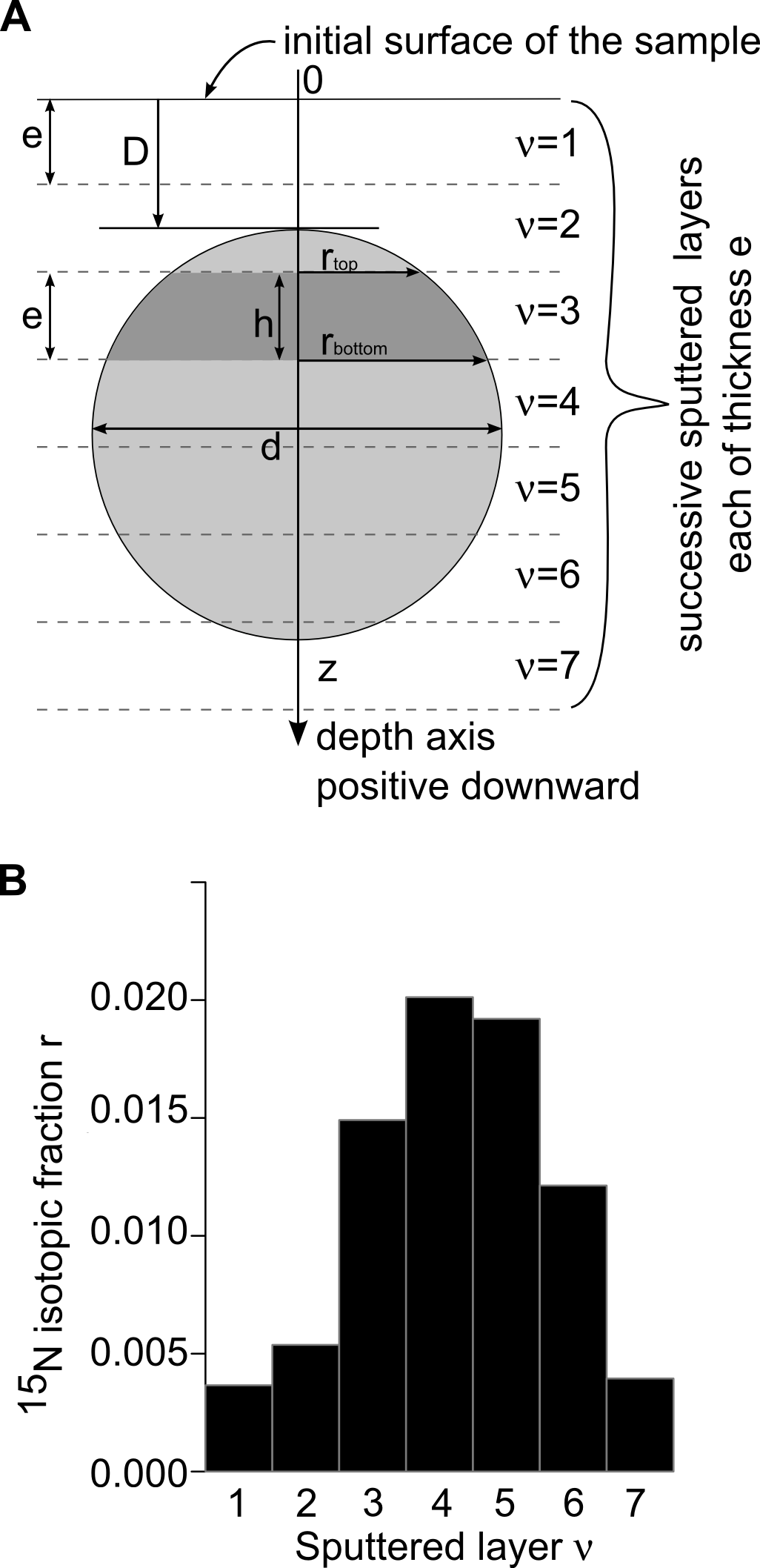

Supplement: Figure S1 — Calculated values of the 15N isotopic fraction in a series of sputtered layers including part of a single enriched protein. (A): Representation of a sequence of sputtered layers. The initial surface of the sample (before sputtering) is chosen as the origin of the depth axis z, positive downward. The successive sputtered layers, each of thickness e, are numbered 1, 2, …ν, … from the initial sample surface. The top of a spherical enriched protein (diameter d) is located at an algebraic distance D from the origin of the depth axis. rtop is the radius of the upper base of the spherical segment of protein in the sputtered layer, rbottom the radius of the lower base and h, the height of the segment (here h = e in each layer except in layers 1, 2 and 7). (B): 15N isotopic fraction in each successive sputtered layer represented in (A). Indeed in layer 1, which does not contain enriched protein, the isotopic fraction is equal to its natural value 0.00366. (TIF) [file pone.0056559.s001.tif]

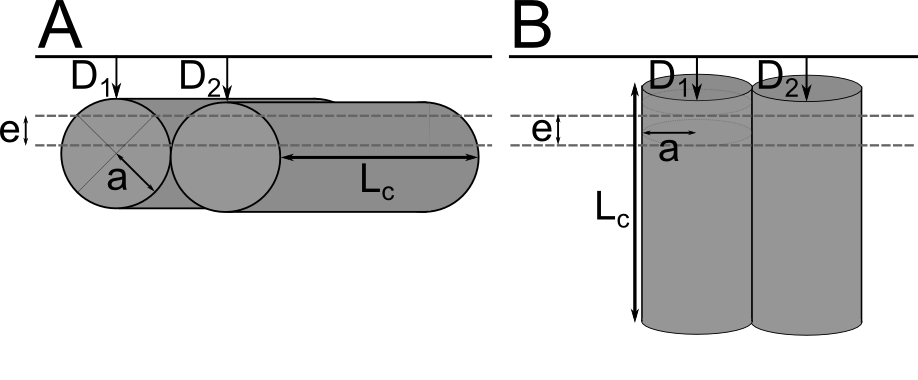

Supplement: Figure S2 — Representation of two cylindrical proteins. (A): the long axis of the proteins is parallel to the initial sample surface. (B): the long axis of the proteins is perpendicular to the initial sample surface. The size of the cylindrical protein is characterized by the length of its long axis Lc and the radius of its section a. The distances of the two proteins relatively to the initial sample surface, D1 and D2, are defined as the algebraic minimal distances between each protein and the initial sample surface. (TIF) [file pone.0056559.s002.tif]

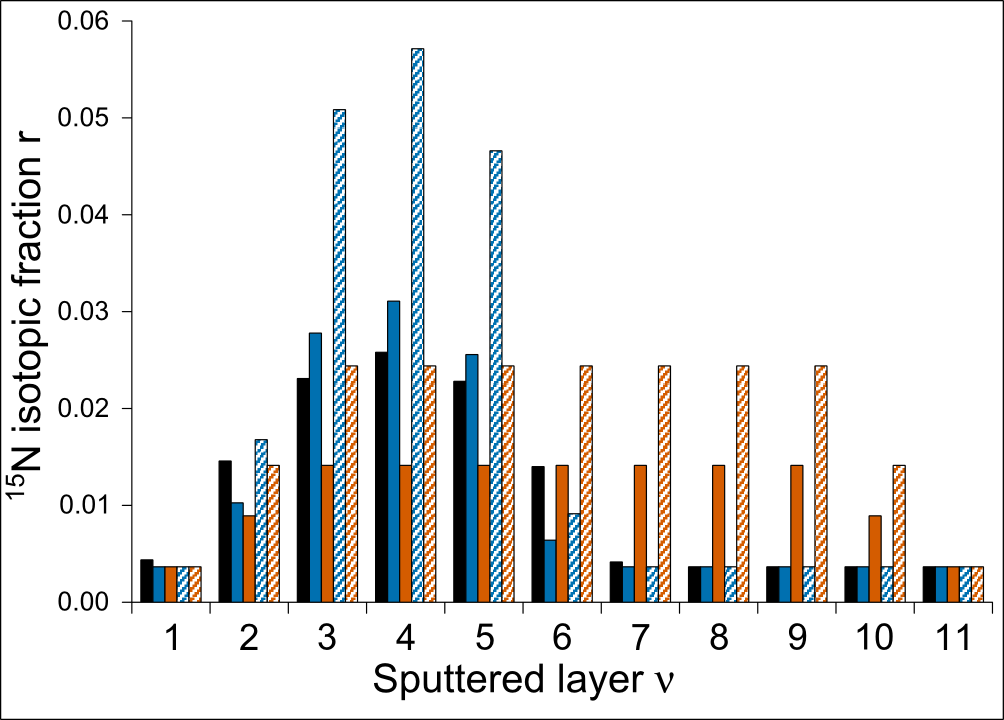

Supplement: Figure S3 — Calculated values of the 15N isotopic fraction in a series of sputtered layers including enriched proteins. These values were calculated for the presence of one (filled bars) or two (shaded bars) 15N-enriched proteins assumed as cylinders with their long axis either parallel (blue bars) or perpendicular (vermilion bars) to the sample surface. The length of the long axis of the cylinder, Lc, is equal to 8 nm and the radius, a, is equal to 1.9 nm. The top of proteins is located at a distance D1 = D2 = 1.5 nm from the sample surface. For comparison, values calculated for the presence of a single spherical 15N-enriched protein (5.6 nm in diameter and located at D = 0.7 nm from the sample surface) are shown (black bars). Each sputtered layer is 1 nm in thickness and (π/4) ×(100)2 nm2 in surface. (TIF) [file pone.0056559.s003.tif]

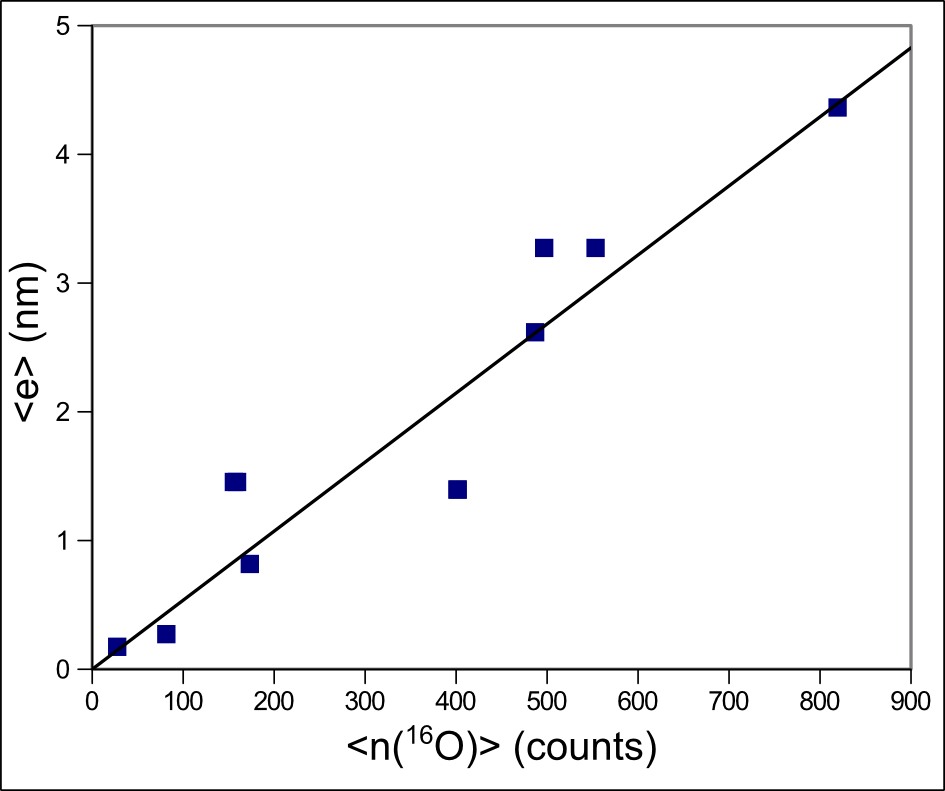

Supplement: Figure S4 — Mean value of the number of 16O secondary ions per pixel as a function of the estimated mean sputtered thickness of the sample. The straight line is the regression line. (TIF) [file pone.0056559.s004.tif]
