# Supplementary material for: 50nm-Scale Localization of Single Unmodified, Isotopically Enriched, Proteins in Cells
Source: PLoS One. 2013 Feb 19;8(2):e56559. doi: 10.1371/journal.pone.0056559 (PMC3576336; doi:10.1371/journal.pone.0056559)
Supplement: Text S1 — Detailed calculations of the expected increase in 15N-isotopic fraction due to the presence of 15N-enriched proteins in the sputtered volume corresponding to a pixel (sputtel). (DOC) [file pone.0056559.s005.doc]

**Supplementary Information 1: Expected increase in the ^15^N isotopic fraction due to the presence of ^15^N-ENRICHED proteins in A SPUTTEL.**

***Case of a single spherical ^15^N-enriched protein***

.

Consider first a sputtel (π/4) ×(100)^2^×4.7 nm^3^. A depth of 4.7 nm was chosen since it is the estimated diameter of a TDG protein treated as a sphere of uniform density of 1.37 g/cm^3^ [1][2]. A primary beam diameter of 100 nm is routinely obtained with a NanoSIMS 50. Assume that this sputtel contains a single ^15^N-enriched TDG protein with, as measured, a ^15^N isotopic fraction [f=^15^N/(^14^N+^15^N)] of 0.98. Hence, this protein contains in average 553×0.98=542 ^15^N atoms and 11 ^14^N atoms. We estimated using the Kjeldahl method that an average COS-7 cell (mean volume 2.3×10^3^ µm^3^) contains 2.89×10^12^ nitrogen atoms, i.e. a nitrogen density ρ_N_ = 2.89×10^12^/2.3×10^12^ = 1,26 at/nm^3^; this result is similar to published values for the human body: 1.34 at/nm^3^, calculated from the data of Frieden [3]. Therefore we estimated that the considered sputtel contained 51459 nitrogen atoms (this represents 100 proteins of a size similar to that of TDG). Taking into account the ^15^N natural abundance (0.00366) these nitrogen atoms include 188 ^15^N isotopes and 51271 ^14^N isotopes. Therefore, if the sputtel contains a single ^15^N-enriched TDG protein, the ^15^N isotopic fraction increases from r_0_=0.00366 to r = (542+188)/(553+51459) = 0.01404 if we consider that the TDG protein has been added to the molecules already present. Therefore the presence of a single TDG protein in the sputtel increases the local CN isotopic fraction by a factor 4.

This calculation assumes that the sputtel contains the entire enriched molecule which, therefore, is also entirely sputtered out. The sputtered thickness in our samples, however, is usually less than 4 nm. In this case, it is only a part of the enriched molecule which is sputtered out as illustrated in Figure S1. We assume that the sputtel has an area of π(L/2)^2^ nm² (L is the effective diameter of the primary beam on the sample surface, e.g. 100 nm) and a thickness e (1 nm in Figure S1) smaller than the protein diameter d (the protein is assumed to be a sphere). A series of successive sputtels is represented in Figure S1A. The initial surface of the sample (before sputtering) is chosen as the origin of the depth axis z positive downward. The successive sputtered layers, each of thickness e, are numbered 1, 2, ...ν, ... from the initial sample surface. The top of the protein is located at an algebraic distance D from the origin of the depth axis (D is negative if the initial sample surface goes through the protein and hence the virtual top of the protein is outside the sample). Indeed, after the sputtering of (ν−1) layers and just before sputtering the ν^th^ layer, the top of the protein is located at a distance D_ν_ = D-(ν−1)e from the sample surface.

Assuming a uniform density of nitrogen atoms in the protein, we calculated the number of ^15^N and ^14^N atoms in each of these successive sputtels (by summing the atoms contained in and out of the volume occupied by the enriched protein). Eventually we calculated the ^15^N isotopic fraction r as a function of D_ν_ using the following equation:

$$r=\frac{\frac{V\times f\times N}{V_{protein}}+r_{0}\times\left(\frac{\pi}{4}\rho_{N}L^{2}e-\frac{NV}{V_{protein}}\right)}{\frac{V\times f\times N}{V_{protein}}+r_{0}\times\left(\frac{\pi}{4}\rho_{N}L^{2}e-\frac{NV}{V_{protein}}\right)+\left(\frac{\pi}{4}L^{2}e-V\right)\rho_{N}\left(1-r_{0}\right)+\frac{\left(1-f\right)NV}{V_{protein}}}$$

where V_protein_=πd^3^/6 is the volume of the entire enriched protein, f the ^15^N isotopic fraction in this protein (0.98 for TDG and 0.94 for RXRα), N the total number of nitrogen atoms in the enriched protein, ρ_N_ the nitrogen density in COS-7 cells (see above), πL²/4 the sputtered area corresponding to a pixel and V (V is a function of D_ν_) the volume of the spherical segment of enriched protein in the sputtered layer given by

$$V=\frac{\pi}{2}\times h\left(r_{top}^{2}+r_{bottom}^{2}+\frac{1}{3}h^{2}\right)$$

where (Figure S1A) r_top_ is the radius of the upper base of the spherical segment, r_bottom_ the radius of the lower base and h, the height of the segment given in Table S1.

**Table S1**: Volumes of enriched protein in the successive sputtered layers 1,2,...,ν,...

| **D_ν_ = D−(ν−1)e** | **h** | **r^2^_top_** | **r^2^_bottom_** | **V** |
| --- | --- | --- | --- | --- |
| e< D_ν_ | 0 | 0 | 0 | 0 |
| 0< D_ν_≤e | e**−**D_ν_ | 0 | **−**(D_ν_**−**e)(d+D_ν_**−**e) | π(e**−**D_ν_)^2^[3d**−**2(e**−**D_ν_)]/6 |
| **−**d+e< D_ν_≤0 | e | **−**D_ν_ (D_ν_+d) | **−**(D_ν_**−**e)(d+D_ν_**−**e) | πe[**−**6D_ν_(D_ν_+d)+3e(d+2D_ν_)**−**2e2]/6 |
| **−**d<D_ν_≤**−**d+e | d+D_ν_ | **−**D_ν_ (D_ν_+d) | 0 | π(D_ν_+d)^2^(d**−**2D_ν_)/6 |
| D_ν_≤**−**d | 0 | 0 | 0 | 0 |

Figure S1B shows the obtained isotopic fraction in each successive sputtered layer represented in Figure S1A. It shows that the value of the isotopic fraction reaches a value close to that calculated above for the sputtering of the entire protein in one step (0.01404) in the 3^rd^ and 6^th^ layers. This fraction can even be higher (0.02 in the case of the layers 4 and 5); this is because the ratio of the relative volume of the protein segment in these thin sputtered layers is higher than when the entire protein is sputtered in one step.

We carried out similar calculations (not shown) in the case of a spherical protein 5.55 nm in diameter (the estimated diameter of a RXRα protein treated as a sphere of uniform density).

In conclusion, even though our calculations are based on a somewhat idealised model, they show that the presence of a single enriched protein in the sputtel increases the local ^15^N isotopic fraction by a factor 4 to 5. Such an increase should be easily measurable if the counting statistics is good enough (see below).

***Case of one or several non-spherical ^15^N-enriched proteins***

Even though numerous proteins can be modelled by a sphere, some cannot and are better shaped by an ellipsoid. In the following we consider the case where the protein is highly extended in one direction and can thus be modelled by a cylindrical rod. We study the two cases where one or two rods are both either parallel or perpendicular to the sample surface (Figure S2). The volume of the model rod is chosen as that of the spherical model protein V_protein_. Therefore the radius, a, of the cylinder section is given by a = (V_protein_/πL_c_)^1/2^; where L_c_ is the length of the cylinder. The distances D_1_ and D_2_ of the 2 proteins from the initial sample surface are defined as shown in Figure S2. The volumes of enriched proteins in the successive sputtered layers are given in Tables S2 and S3 for a cylinder parallel or perpendicular to the sample surface, respectively. The sputtered layers are assumed of equal thickness. The ^15^N-isotopic fraction is calculated as explained above in the case of a spherical protein.

**Table S2**: Volumes of enriched protein in the successive sputtered layers 1,2,...,ν,...(case of cylinder parallel to the sample surface)

| **D_ν_ = D−(ν−1)e** | **V*** |
| --- | --- |
| D_ν_>e | 0 |
| 0<D_ν_≤e | (L_c_a^2^/2)[ω **−**sin ω] |
| **−**2a+e<D_ν_≤0 | (L_c_a^2^/2)[(β **−** sin β) **−** (ω **−** sin ω)] |
| **−**2a<D_ν_≤**−**2a+e | (L_c_a^2^/2)[π **−** (β **−** sin β)] |
| D_ν_≤**−**2a | 0 |

*: β = 2arccos((a+ D_ν_ )/a) and ω = 2arccos((a+ D_ν_**−**e)/a)

**Table S3**: Volumes of enriched protein in the successive sputtered layers 1,2,...,ν,...(case of cylinder perpendicular to the sample surface)

| **D_ν_ = D−(ν−1)e** | **V** |
| --- | --- |
| D_ν_>e | 0 |
| 0<D_ν_≤e | πa^2^[e **−** D_ν_] |
| **−**L_c_+e<D_ν_≤0 | πa^2^e |
| **−**L_c_<D_ν_≤**−**L_c_+e | πa^2^[L_c_ + D_ν_] |
| D_ν_≤-L_c_ | 0 |

Figure S3 shows the calculated ^15^N isotopic fraction for 1 or 2 cylindrical proteins either parallel or perpendicular to the sample surface. In this example we chose a reasonable length L_c_ of 8 nm for the cylinder (hence its radius is 1.9 nm). The figure also shows the ^15^N isotopic fractions obtained with a spherical protein of same volume. Our calculations show that, as in the case of a spherical protein, the presence of a single cylindrical enriched protein in the sputtel increases substantially the local ^15^N isotopic fraction. Depending on the orientation of the cylinder relatively to the sample surface (perpendicular to parallel) the increase is by a factor 4 to 8.

Figure S3, however, shows that a similar increase in isotopic fraction can be obtained with a single spherical protein or 2 perpendicular cylindrical proteins at least for the 5 first sputtered layers. Therefore, in this particular case, there is a risk to wrongly conclude to the presence of 2 proteins whilst only 1 is present (or the converse). Nevertheless if the shape (actual protein dimensions) is known it can be discriminated between the two models (1 sphere or 2 rods) and therefore the real number of proteins that is being sputtered can be determined. Indeed, even if the shape of the protein is unknown, the complete sputtering of the protein (11 layers in the case of Figure S3) clearly permits to distinguish between the 1 sphere or 2 rods models (see layers 6 to 11 in Figure S3).

In Figure 2B we have shown that the sputtering of 2 spherical proteins with adequate distances relatively to the sample surface gives an increase of the isotopic fraction almost constant for 7 layers as in the case of 2 cylindrical proteins perpendicular to the surface (Figure S3). Moreover a similar increase in ^15^N isotopic fraction can be obtained in successive sputtered layers in the case of 2 spherical proteins (Figure 2C) and 2 cylindrical proteins parallel to the sample surface. Hence, even though the real shape of the protein is unknown, the determination of the number of sputtered proteins is reliable.

***Supplementary references***

1. Gekko K, Noguchi H (1979) Compressibility of globular proteins in water at 25 degree C. J Phys Chem 83: 2706–2714. doi:10.1021/j100484a006.

2. Squire PG, Himmel ME (1979) Hydrodynamics and protein hydration. Arch Biochem Biophys 196: 165–177.

3. Frieden E (1972) The chemical elements of life. Scientific American 227: 52–60.
