# Supplementary material for: 50nm-Scale Localization of Single Unmodified, Isotopically Enriched, Proteins in Cells
Source: PLoS One. 2013 Feb 19;8(2):e56559. doi: 10.1371/journal.pone.0056559 (PMC3576336; doi:10.1371/journal.pone.0056559)
Supplement: Text S2 — Algorithm for the determination of the probability distribution law of the background 15N-isotopic fraction. (DOC) [file pone.0056559.s006.doc]

**Supplementary Information 2: Determination of the Probability distribution law for the background ^15^N isotopic fraction.**

***Selection of the set of pixels to be analysed****.*

Two sets of pixels are excluded from the analysis.

1): pixels of the 3 first and last columns and rows are not taken into account in the algorithm. The reason for this is to avoid assigning unjustified values to the “pixels” out of the image field as would be required if the process used in the algorithm (see below) was applied to the pixels of these rows or columns. Therefore the sole pixels (i,j) that are analysed, are those in a square (X**−**6)×(X**−**6) domain defined by [rows ; columns]=[4,X**−**3 ; 4,X**−**3] where X, as already stated, is the number of rows and columns in the original image. Hence, for pixels outside this domain, it cannot be decided whether the possible finding of a high isotopic fraction is due to the presence of enriched molecules or to statistical fluctuations in the counting. It should, however, be noted that in high resolution imaging (small field of view) there is few or even none cell material near the edges of the image; thus generally there is not lost of information.

2): areas that do not contain any biological material (embedding resin) are also excluded from the analysis via a thresholding process based on the n_ij_(^12^C^14^N) values. Indeed, the embedding resin contains in principle only traces of nitrogen (impurities) whereas cell areas have high nitrogen content. Hence, pixels having values below a user-chosen threshold are excluded from the analysis whereas pixels having values above are analyzed. Finally Y_sel_ is the total number of analysed pixels.

***Determination of the parameters $\lambda_{\text{ij}}^{14}$and* $\lambda_{\text{ij}}^{15}$ *of the Poisson probability distribution laws for ^12^C^14^N and ^12^C^15^N background counts.***

The determination of the probability distribution law for the isotopic fraction r_ij_ requires for each pixel (i,j) the determination of the Poisson distribution laws for the background values of n_ij_(^12^C^14^N) and n_ij_(^12^C^15^N) in the imaged sample. Biological samples are highly heterogeneous, even at the level of a 100 nm which is the typical size of the sputtered area corresponding to the pixel. Hence the count number n_ij_(^12^C^14^N) is the sum on the n^k^_ij_(^12^C^14^N) secondary ions coming from the different heterogeneous structures k=1, 2,... contained in the sputtered volume corresponding to this pixel. Thus n_ij_(^12^C^14^N) = n^k^_ij_(^12^C^14^N) is the sum of Poisson random variables and therefore is also a Poisson random variable. The parameters λ_ij_(^12^C^14^N) and λ_ij_(^12^C^15^N) of the Poisson distribution laws associated to the pixel (i,j) (noted simply λ^14^_ij_ and λ^15^_ij_) are the sum of the parameters λ^14^_ij_(k) and λ^15^_ij_(k), respectively, of the different heterogeneous structures. The aim here is to estimate these λ^14^_ij_ and λ^15^_ij_ Poisson parameters for each selected pixel.

Consider, first the case of ^12^C^14^N. Even though there are heterogeneities at the macromolecular level, the cells have often a homogeneous *elemental* composition at the level of a µm. Therefore, as the SIMS averages the number of secondary ions extracted from the different structures in the sputtel, the pixels in the immediate vicinity of a pixel (i,j) have the same λ^14^_ij_. We assume here that this is the case for the 48 pixels in the (7×7) square matrix of pixels centred on the pixel (i,j). We determined empirically (see also the simulation experiment described below) that a (7×7) matrix was the best compromise between precision (large matrix) and adequacy to the homogeneity hypothesis (small matrix). A first estimation of λ^14^_ij_{1} is simply n_ij_(^12^C^14^N). To improve this estimation we increase or decrease the λ^14^_ij_ value by 1 using the equation:

λ^14^_ij_{v} = λ^14^_ij_{1} ± (v**−**1)×1 with v= 1, 2, 3,...

For each value λ^14^_ij_{v} at the step v, we calculate the sum of the Poisson probabilities to obtain the measured n_pq_(^12^C^14^N) values with p∈[i**−**3;i+3], q∈[j**−**3;j+3], i.e. the pixels of the square matrix. We choose as the best estimate of λ^14^_ij_, the value λ^14^_ij_{v} which maximizes this sum.

When the pixel (i,j) is in a transition area between two large cell distinct structures, the homogeneity hypothesis may fail. In the case where the matrix contains two classes of pixels, corresponding to two distinct homogeneous areas, the iterative improvement process described above yields 2 maxima for the sum of the Poisson probabilities. Indeed the chosen λ^14^_ij_ is that for which the probability to obtain the measured n_ij_(^12^C^14^N) is maximum.

Eventually a test of Kolmogorov-Smirnov is carried out, to a risk of 1 %, to verify that the central pixel (i,j) is not the sole in the matrix to be compatible with the Poisson law of determined parameter λ^14^_ij_. If it is the case, the determined λ^14^_ij_ is unreliable; this is probably due to the extreme heterogeneity of the area or to an uncontrolled counting artefact. Thus such pixel is discarded from the analysis (the pixel is displayed in black in the processed image).

Finally, we used a simulation experiment to test the efficacy of the algorithm to improve the λ determination. Starting with a uniform image, i.e. with pixels of the same value n, we added a Poisson “noise” (λ=mean= variance = n) to each pixel. As explained above, the first estimation of the Poisson parameter, λ_ij_{1}, is simply n_ij_. Therefore the relative dispersion of this first estimation of the Poisson parameter in the image is given by the ratio of the standard deviation to n (here trivially $1/\sqrt{n}$). After running the algorithm, a decrease (ideally to zero) of this relative dispersion is expected. For example with n=500 (a typically value obtained in ^12^C^14^N counting in our samples) the relative dispersion for the first estimation of λ is 0.0447. After running the algorithm the mean is still very close to n and the relative dispersion is: 0.0180, 0.0084 and 0.0068 using a (5×5), (7×7) and (9×9) matrix respectively. Indeed as expected there is a substantial improvement of the λ determination when running the algorithm; moreover the benefit of increasing the matrix size beyond (7×7) is only moderate compared to the drawback of the quadratic increase in the calculation time. In the following we thus limited the matrix size to (7×7); this choice is additionally justified by the fact that increasing the matrix size increases the risk of failure of the homogeneity hypothesis in real biological samples.

Consider now the case of ^12^C^15^N. Due to the low values of the n_ij_(^12^C^15^N) the determination of λ^15^_ij_ using the algorithm will be imprecise; we checked this conclusion using the same simulation as above but with low values of n (not shown). Moreover the n_ij_(^12^C^15^N) value cannot be a measure of the background when one or several enriched molecules are present in the sputtel. Furthermore in the case where the central pixel is enriched whilst none of the pixels in its vicinity is enriched, the final step of the algorithm (Kolmogorov-Smirnov test) likely will discard this pixel and worse, if a second (or few) pixel in the vicinity is enriched, the algorithm will give a λ^15^_ij_ value which is not characteristic of the background. Therefore for all these reasons we estimate the Poisson parameters for ^12^C^15^N ions as being simply related to that of ^12^C^14^N by:

where instead of using the value of the natural ^15^N fraction found in literature (r_0_), we used <r>, the value we measured by averaging the ^15^N fraction of all the pixels analysed in the studied image (*i.e.* the pixels selected after the initial thresholding). The presence of a few enriched pixels compared to the few tens of thousands of unenriched pixels, does not significantly alter this determination. Moreover, this average value takes into account deviations in the measured isotopic fraction due to instrumental settings or SIMS isotopic fractionation. That said we always found values <r> very close to r_0_ (not shown).

In conclusion the probabilities P^14^_ij_[n_14_] or P^15^_ij_[n_15_] to have the count numbers n_14_ = n_ij_(^12^C^14^N) or n_15_ = n_ij_(^12^C^15^N) for the ^12^C^14^N and ^12^C^15^N ions respectively, are given by the Poisson laws

or

***Determination of the probability distribution law for the background ^15^N isotopic fraction.***

Consider now couples of putative integer values n_14_ and n_15_ for the count numbers of the ^12^C^14^N and ^12^C^15^N ions extracted from a volume corresponding to a pixel. These values correspond to ^15^N isotopic fractions equal to r = n_15_/( n_15_ + n_14_) and the probability of their simultaneous counting is

P[n_14_ ****n_15_] = P^14^[n_14_]×P^15^[n_15_]

There is a number of couples of count numbers, noted n_14_(r) and n_15_(r), that give the same isotopic fraction r. Therefore the probability of measuring a ^15^N isotopic fraction equal to r is

P(r) = ∑_r_ P^14^[n_14_(r)]×P^15^[n_15_(r)]

where the notation ∑_r_ means that the summation is extended to all the couples of possible values n_14_(r) and n_15_(r) giving the same r. In practice the probabilities P_ij_[n_14_ ****n_15_] together with the corresponding values r_ij_(n_14_, n_15_) were calculated for the positive integers n_14_ and n_15_ in the range and respectively. This truncation limits the number of calculations and thus the computation time; we checked that

0.999999 < ∑ P^14^_ij_[n_14_]×P^15^_ij_[n_15_] < 1

where the notation ∑ means that the summation is extended to all the couples of values n_14_ and n_15_ in this range. This shows that the relative truncation error of our computation method is less than 10^-6^. We thus obtained, for each pixel (i,j), a table T_ij_=(n_14_; n_15_; r; P_ij_[n_14_ ****n_15_]). We then arranged the r values in a series of 10^5^ classes and calculated the corresponding probabilities by summation of the P[n_14_ ****n_15_] in each class. This gave us, for each pixel (i,j) an estimation of the probability distribution function P_ij_(r) for the background ^15^N isotopic fraction. Therefore the cumulative probability to obtain a ^15^N isotopic fraction greater than the measured r_ij_ (p-value) is calculated for each pixel with:

(p-value)_ij_ = 1–

This p-value is the probability that the measured r_ij_ is an observation of the background ^15^N isotopic fraction. Hence, the lower the p-value the lower the probability that this pixel is unenriched and indeed the higher the probability that this pixel is enriched.
