# Supplementary material for: 50nm-Scale Localization of Single Unmodified, Isotopically Enriched, Proteins in Cells
Source: PLoS One. 2013 Feb 19;8(2):e56559. doi: 10.1371/journal.pone.0056559 (PMC3576336; doi:10.1371/journal.pone.0056559)
Supplement: Text S3 — Method for the determination of the sputtered thickness. (DOC) [file pone.0056559.s007.doc]

**SUPPLEMENTARY INFORMATION 3: Estimation of the sputtered thickness**

The determination of the number of sputtered proteins depends on the thickness of sputtered layers compared to the size of the enriched proteins. The mean sputtered thickness on the entire field of view, <e> in nm can be calculated with the classical SIMS formula (*e.g.* [1]):

$$<e> =7.093\times10^{-5}SX^{2}i\tau/\delta^{2}$$

in which S is the sputtering efficiency (i.e. the number of sputtered particles per incident Cs^+^ ion) here we chose 5 [1]; X² is the number of pixels in the image; δ×δ is the field of view in µm²; i is the primary current intensity in pA; τ is the dwell time in ms and the numerical factor includes the total atomic density of the samples 8.8×10^10^ atoms/µm^3^. However, local differences in the sputtered thickness can result from topographic (roughness of the sample surface, etc.) or instrumental (non uniform primary ion density on the imaged field, etc.) parameters. To take into account these differences and obtain a value of the sputtered thickness pixel by pixel e_ij_ we used the count number n_ij_(^16^O) for the ^16^O secondary ions. Indeed we observed that there was a reasonably good correlation between <e> calculated using the above formula and <n(^16^O)>, the mean value of the n_ij_(^16^O) in the image. Figure S4 shows the result obtained with 10 independent control images (series of sample preparations carried out for RXRα imaging). The near uniformity of the oxygen concentration in the resin embedded biological samples justify a linear regression, that gave

<e> = 5.4×10^-3^ <n(^16^O)>

with a regression coefficient of 0.957. For the series of sample preparations carried out for TDG imaging the regression law was

<e> = 1.66×10^-2^ <n(^16^O)>

with a regression coefficient of 0.907. This difference is likely due, in the case of preparation of TDG samples, to the use of a different batch of LR White resin and to a better degassing before polymerisation. We then assumed that these statistical laws were an estimate for the deterministic local laws giving the local sputtered thickness e_ij_ (i.e. for the pixel (i,j)) as a function of the local ^16^O counts, n_ij_(^16^O), i.e: e_ij_ = k× n_ij_(^16^O) with k equal 5.4×10^-3^ and 1.66×10^-2^ respectively for preparations carried out for RXRα and TDG imaging. We further justified this assumption using control LR White resin sections which were SIMS imaged and the corresponding sputtered areas of which were imaged using AFM (Nanoscope III, Digital instruments, Santa Barbara, CA) in contact mode with a piezoelectric J scanner. Some SIMS images of n(^16^O) showed a gradient that we attributed to a non uniform sputtering. This was confirmed by the AFM measurement of the depth in a series of locations in the sputtered area. Moreover the AFM-measured depth was consistent with the calculated values using the above formulas (data not shown). It should be noted, however, that in the case of a very weak sputtering obtained with a low primary beam intensity and dwell time, the sputtered thickness calculated using these formulas can be as low as 0.05 nm. This clearly means that in the sample area corresponding to a pixel the molecules are unevenly fragmented and that e_ij_ is simply the numerical value such as πe_ij_L²/4 is the sputtered volume corresponding to the pixel. In this case, scarcely encountered in our experiments, e_ij_ must be viewed as an operational thickness.

***Supplementary references***

1. Lechene C, Hillion F, McMahon G, Benson D, Kleinfeld AM, et al. (2006) High-resolution quantitative imaging of mammalian and bacterial cells using stable isotope mass spectrometry. J Biol 5: 20. doi:10.1186/jbiol42.
